# The Influence of Training with an Evaluation Mask on Physiological Adaptations in a Recreational Athlete

**DOI:** 10.3390/jfmk11010054

**Published:** 2026-01-27

**Authors:** Marko Kunac, Petar Šušnjara, Danijela Kuna

**Affiliations:** 1 Department for Common Subjects, University of Applied Sciences “Lavoslav Ružička” in Vukovar, 32000 Vukovar, Croatia; mkunac@vevu.hr; 2 Faculty of Kinesiology, University of Zagreb, 10000 Zagreb, Croatia; 3 Faculty of Kinesiology Osijek, Josip Juraj Strosssmayer, University of Osijek, 31000 Osijek, Croatia; psusnjara@kifos.hr

**Keywords:** elevation training mask, high-intensity interval training, oxygen saturation, hematological adaptations, recreational athletes

## Abstract

**Background:** Innovative training strategies aimed at improving physiological efficiency are of growing interest in kinesiology and sports performance. Elevation training masks (ETMs) offer a practical means of inducing hypoxia-like stress. However, evidence of their effectiveness in recreationally active populations remains limited. This pilot study examined the efficiency of a five-week progressive ETM protocol combined with high-intensity interval training (HIIT) in eliciting physiological, hematological, and body-composition adaptations relevant to endurance performance. **Methods:** Nine recreationally active men completed a five-week intervention consisting of three treadmill-based sessions per week: one weekly incremental Conconi test and two structured aerobic–anaerobic HIIT sessions performed with an ETM. Mask resistance was progressively increased to simulate altitudes of approximately 900–3600 m. Hematological variables (erythrocytes, hemoglobin, hematocrit, erythrocyte indices, leukocytes, and platelets), body composition, maximal heart rate (HRmax), and peripheral oxygen saturation (SpO_2_) were assessed pre- and post intervention. Data were analyzed using paired-sample *t*-tests and repeated-measures ANOVA, with effect sizes reported (Cohen’s d, ω^2^). **Results:** A significant main effect of time on SpO_2_ was observed (F(1, 8) = 130.61, *p* < 0.001, ω^2^ = 0.69), along with a significant effect of training week (F(4, 32) = 17.41, *p* < 0.001, ω^2^ = 0.43), and a significant Time × Week interaction (F(4, 32) = 15.20, *p* < 0.001, ω^2^ = 0.42), indicating progressively greater post-exercise oxygen desaturation with increasing simulated altitude. Significant post-intervention increases were found in erythrocyte count, hemoglobin concentration, and hematocrit (*p* ≤ 0.009, d = 1.15–1.55), alongside increases in mean corpuscular volume and mean corpuscular hemoglobin. Platelet count increased significantly (*p* = 0.001, d = 1.68), while leukocyte values remained unchanged (*p* > 0.05). Body mass index (*p* = 0.049, d = 0.77) and body fat percentage (*p* = 0.012, d = 1.08) decreased following the intervention. HRmax tended to be lower at higher simulated altitudes. **Conclusions:** A five-week progressive ETM-HIIT protocol efficiently induced hematological and body-composition adaptations associated with improved oxygen transport and metabolic efficiency in recreationally active men. These findings support ETM-based training as an accessible strategy for enhancing physiological efficiency in endurance-oriented kinesiology practice, warranting confirmation in larger randomized controlled studies.

## 1. Introduction

In recent decades, methods for improving aerobic fitness and sports performance have increasingly focused on optimizing physiological adaptations through various training modalities and technological aids. One innovative approach involves the use of elevation training masks (ETMs), designed to restrict airflow during breathing, thereby creating conditions that mimic hypoxic stress similar to altitude training [1]. It should also be emphasized that ETMs serve the training respiratory muscles by increasing breathing resistance, which can induce mild hypoxemia similar to functional hypoxia during exercise. Scientific articles show that they do not simulate true altitude, but rather act like respiratory muscle training devices, improving ventilatory thresholds and endurance performance [1]. In recent years, elevation masks have gained considerable popularity as they aim to replicate the continuous exposure to altitudes above 1500 m, which has been shown to enhance oxygen transport capacity by increasing erythrocyte count through compensatory mechanisms and elevated erythropoietin secretion [2,3]. Consequently, endurance athletes often train and live in hypoxic conditions to achieve better competitive results [4,5].

Generally, hypoxic conditions reduce the partial pressure of oxygen (pO_2_) in the blood, which peritubular cells in the kidney detect and activate HIF-1α, promoting the expression of the erythropoietin (EPO) gene [6,7]. Elevated EPO concentration stimulates the differentiation and proliferation of erythroid progenitor cells in the bone marrow, increasing the number of erythrocytes and myoglobin in muscles for better oxygen delivery. HIF-1α further regulates adaptive changes such as angiogenesis (via VEGF), a shift to anaerobic metabolism, and improved sports endurance in normoxia [8,9].

Although ETM is primarily designed for respiratory muscle training, research has shown that its use can achieve significant adaptive effects in the form of increased hematocrit, erythropoietin concentration, hemoglobin, and hematocrit and improved physical endurance after 6 weeks of intermittent ETM training, which are similar to traditional altitude training [10,11,12].

Furthermore, adaptive changes from hypoxic training encompass other systemic adjustments in the body, including cardiovascular, metabolic, immune, and vascular mechanisms. At the same time, mild hypoxemia stimulates carotid chemoreceptors, activating vagal tone, and reducing sympathetic activity, which ultimately inhibits an unnecessary increase in HR, and thus the heart becomes more economical because the lungs compensate for hypoxic conditions. It is certainly worth emphasizing improved organism oxygenation as well as fatty acid mobilization, where hypoxemic conditions promote fatty acid oxidation, which was recorded in our study in the form of reduced body mass, though these results should be taken with a dose of caution [13,14,15].

Therefore, it can be concluded that respiratory muscle training using ETM can cause “functional hypoxia” conditions in the body and thereby improve specific sports performance. However, there are controversies between classic altitude training and training with altitude masks that arise from the difference between true hypoxia (reduced pO_2_) and mechanical resistance to breathing (reduced FiO_2_ but lower alveolar pO_2_), which leads to different adaptations [16,17].

Due to the lack, ambiguity, and inconsistency of research on the effects of elevation masks in recreational athletes, the aim of this study is to investigate the impact of training with elevation masks on key blood parameters (erythrocyte, hemoglobin, hematocrit) and oxygen saturation (SpO_2_) during a training protocol in recreational athletes.

## 2. Materials and Methods

### 2.1. Participants

Nine recreationally active male participants took part in this study. They had regularly engaged in structured aerobic–anaerobic endurance programs, primarily focused on running and cycling, for at least one year prior to the study. Their previous physical activity consisted mainly of continuous moderate-intensity aerobic work with occasional high-intensity interval segments, typical of recreational endurance programs. The average age of the participants was 21 ± 8 years, and all were in good general health. Inclusion criteria included the absence of known cardiovascular, respiratory, metabolic, or hematological diseases, as well as regular training at least three times per week during the previous 12 months. Additionally, participants had no prior experience with elevation masks or similar altitude-simulation devices. Exclusion criteria included smoking, use of medications or dietary supplements that could affect hematological parameters or cardiovascular responses to exercise, acute infections during the study period, and any injuries or health conditions that might limit safe participation in testing or training protocols. Given the small number of participants and the exploratory nature of the study, it was designed as a pilot investigation to preliminarily assess acute and short-term physiological responses to elevation mask use under simulated altitude conditions. All participants were fully informed about the study’s objectives, procedures, and potential risks and provided written informed consent prior to participation. The study was conducted in accordance with the Declaration of Helsinki, and the research protocol was approved by the competent ethics committee (classification number: 602-01/24-02/01, reference number: 2196/115/01-24-172, date: 11.11.2024).

### 2.2. Experimental Design

The study lasted a total of five weeks, during which participants were exposed to progressively modified simulated altitude conditions using an elevation training mask with different respiratory resistance filters.

One session per week consisted of a Conconi incremental exercise test, while the remaining two weekly sessions involved structured aerobic–anaerobic treadmill training with the ETM. Additional sessions were designed as a combination of moderate- to high-intensity interval work and continuous submaximal exercise, aimed at inducing controlled hypoxic respiratory stress without maximal exhaustion. The Conconi test was performed once per week to provide a standardized assessment of responses to progressive loading and to monitor changes in peripheral oxygen saturation (SpO_2_) throughout the intervention, while avoiding excessive testing burden on participants.

Simulated altitude conditions were progressively increased over the five consecutive weeks as follows: the first week of testing was conducted without the mask, while in subsequent weeks, respiratory filters simulating approximate altitudes of 900 m, 1800 m, 2700 m, and 3600 m were used.

### 2.3. Protocol of the Incremental Exercise Test

The Conconi incremental exercise test was performed on a treadmill at a constant 1.5° incline. Participants began walking at 3.0 km/h, after which the speed was increased by 0.5 km/h every 30 s until voluntary exhaustion or an inability to maintain the prescribed speed [18]. During the test, heart rate was continuously monitored using a chest heart rate sensor (Polar H10, Polar Electro, Vanta, Finland) to control exercise intensity and ensure maximal effort. Maximal heart rate (HRmax) was defined as the highest recorded value immediately before test termination and was used as an additional indicator of comparability of exercise intensity across measurements. Peripheral oxygen saturation (SpO_2_) was measured non-invasively using a pulse oximeter (ChoiceMMed MD300C29, ChoiceMMed Co., Ltd., Beijing, China) immediately before the test (pre-test) and immediately after its completion (post-test) and served as the primary physiological outcome measure of this study.

### 2.4. Anthropometric and Body-Composition Variables

Body composition assessment was performed using the TANITA MC-780MA (Tanita Health Equipment H.K. Ltd., Hong Kong, China) body composition analyzer. Measured variables included body mass and height, from which body mass index (BMI) was calculated.

Additionally, fat percentage (FAT P), absolute fat mass (FAT M), fat-free mass (FFM), visceral fat level (VFatL), bone mass (BoneM), basal metabolic rate (BMR), metabolic age (MetaAge), and phase angle (Phase) were analyzed. Body height was measured using a standardized Seca stadiometer type 700 (Seca Gmbh & co., Hamburg, Germany).

### 2.5. Hematological Variables

Venous blood samples were collected in the morning after an overnight fast, following 15 min of rest in a seated position, one day before the start of the intervention and 24 h after the final testing session.

The following hematological parameters were analyzed: erythrocyte count (ERIT), hemoglobin concentration (HEMO), hematocrit (HEMA), mean corpuscular volume (MCV), mean corpuscular hemoglobin (MCH), mean corpuscular hemoglobin concentration (MCHC), red cell distribution with (RDW), leukocyte count (LEUK), platelet count (TROMB), and mean platelet volume (MPV). Analyses were performed at the Laboratory for Laboratory and Transfusion Medicine, National Memorial Hospital “Dr. Juraj Njavro” Vukovar. Blood samples were collected one day before the start of this study and 24 h after the final testing.

### 2.6. Statistical Analysis

Skewness and kurtosis were calculated to assess approximate normality. Normality was evaluated based on skewness and kurtosis values; values within ±1.0 were considered indicative of an approximately normal distribution, while values up to ±2.0 were accepted as permissible given the small sample size and exploratory nature of the study. Due to the sample size, formal normality tests were not applied, and assessment relied on a combination of descriptive indicators and visual inspection of distributions. Differences between baseline and final measurements were analyzed using paired-sample *t*-tests. In addition to statistical significance, effect sizes were calculated using Cohen’s d to assess the practical importance of observed changes (d ≈ 0.20 small, d ≈ 0.50 medium, d ≥ 0.80 large). Changes in maximal heart rate (HRmax) and peripheral oxygen saturation (SpO_2_) during the Conconi test across the five-week intervention were analyzed using repeated-measures ANOVA. Within-subject factors were time of measurement (pre–post) and intervention week (1–5). For each effect, F-values, significance levels, and effect sizes expressed as ω^2^ were calculated. Statistical significance was set at *p* < 0.05. However, given the pilot nature of the study and small sample size, interpretation of the results primarily focused on the direction of changes and effect sizes rather than solely on statistical significance. Data processing was performed using JASP software (version 0.95.2).

## 3. Results

**Figure 1:**
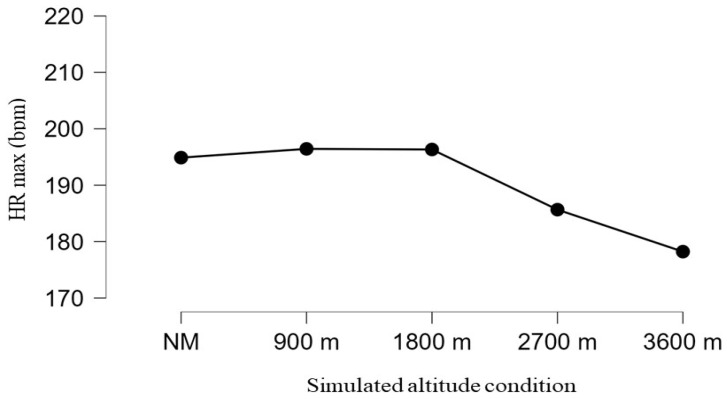
Changes in maximal heart rate (HRmax) across simulated altitude conditions.

Figure 1 illustrates maximal heart rate (HRmax) values achieved during the Conconi incremental exercise test under different simulated altitude conditions and indicates a differentiated physiological response depending on the degree of respiratory loading. In conditions without the mask (NM) and at lower simulated altitudes (900 m and 1800 m), similar HRmax values were recorded, suggesting preserved ability to achieve maximal cardiovascular effort despite a moderate reduction in oxygen availability.

In contrast, at higher simulated altitudes (2700 m and 3600 m), there was a pronounced decrease in HRmax, indicating changes in cardiovascular response associated with hypoxic loading and limitations in achieving maximal effort. This decline most likely reflects the combined effect of reduced oxygen availability, increased ventilatory load, and earlier onset of fatigue, which may limit the ability to reach true maximal workload. However, given repeated exposure to simulated altitude conditions over several weeks, the possibility of an early adaptive cardiovascular response cannot be entirely excluded, which may manifest as a lower maximal heart rate at the same or similar relative workload.

**Figure 2:**
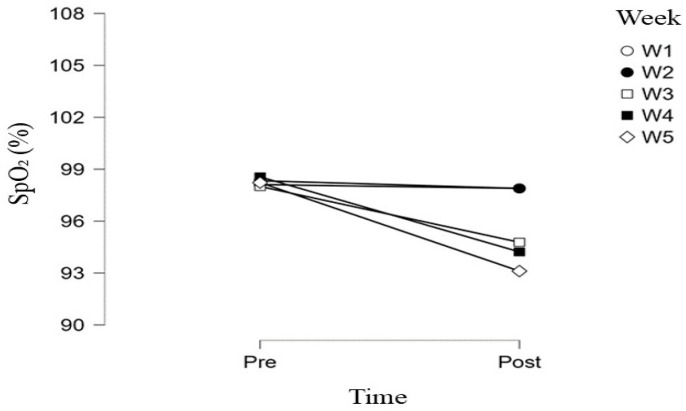
Changes in arterial oxygen saturation (SpO_2_) during the Concconi test across five weeks of simulated hypoxic training.

Changes in heart rate were complemented by alterations in peripheral oxygen saturation (SpO_2_), as illustrated in Figure 2. Pre-test SpO_2_ values remained stable and high (≈98%) across all five weeks, with minimal variability. In contrast, post-test SpO_2_ values showed a progressive decline throughout the intervention, particularly from the third week onward.

As shown in Figure 2, differences between pre- and post-test SpO_2_ measurements were minimal during the first two weeks, whereas progressively greater post-exercise reductions were observed in the third, fourth, and fifth weeks. The lowest mean post-test SpO_2_ values were recorded in the fifth week (≈93%), accompanied by increased inter-individual variability.

Overall, changes in HRmax and SpO_2_ indicate that the use of an elevation mask with progressively modified respiratory filters leads to gradual increases in physiological load, with SpO_2_ changes appearing as a more sensitive and direct indicator of hypoxemic stress compared to the maximal cardiac response.

Repeated-measures ANOVA revealed a statistically significant main effect of time on SpO_2_ (F(1, 8) = 130.61, *p* < 0.001, ω^2^ = 0.69), indicating systematically lower post-test SpO_2_ values compared to pre-test, regardless of measurement week. A significant main effect of week was also found (F(4, 32) = 17.41, *p* < 0.001, ω^2^ = 0.43), pointing to progressive SpO_2_ changes over the five-week program.

Furthermore, a significant Time × Week interaction (F(4, 32) = 15.20, *p* < 0.001, ω^2^ = 0.42) showed that the difference between pre- and post-test SpO_2_ values increased with progression of the intervention, with the most pronounced changes recorded in the final weeks. These findings confirm that the progressive modification of respiratory filters in the ETM, simulating different altitudes, results in targeted and progressive induction of a hypoxemic response during exercise.

In addition to acute hypoxic responses, descriptive analysis of anthropometric and body-composition variables (Table 1) revealed moderate but consistent changes between baseline and final measurements. A slight decrease in body mass index was observed from baseline to final measurement (24.20 ± 3.48 vs. 23.77 ± 3.36), accompanied by a similar trend in fat tissue indicators. Fat percentage and absolute fat mass showed a moderate decline in mean values, while visceral fat level was also slightly lower at final measurement. Fat-free mass and bone mass exhibited a minor decrease in mean values, basal metabolic rate showed a slight reduction, and metabolic age remained largely unchanged. Value ranges and skewness/kurtosis indicators suggest that the overall variability of most anthropometric and compositional parameters did not change substantially between measurements.

Paired-sample *t*-test analysis (Table 2) further confirmed that some of the observed changes were statistically significant. Significant reductions were found in body mass index (*p* = 0.049, d = 0.77) and fat percentage (*p* = 0.012, d = 1.08), with medium to large effect sizes. Fat-free mass and bone mass also showed statistically significant changes (*p* ≤ 0.016) with large effect sizes, whereas changes in visceral fat and metabolic age did not reach statistical significance, although moderate effects were observed.

The most pronounced changes were recorded in hematological parameters of the erythroid lineage. Erythrocyte count, hemoglobin concentration, and hematocrit significantly increased between baseline and final measurements (*p* ≤ 0.009), with large to very large effect sizes (d = 1.15–1.55). Erythrocyte indices also showed consistent changes, with mean corpuscular volume and mean corpuscular hemoglobin significantly increasing, while mean corpuscular hemoglobin concentration remained stable. Red cell distribution width exhibited a statistically significant change with a large effect, indicating alterations in the heterogeneity of the erythrocyte population.

Leukocyte values showed no significant changes during the intervention, suggesting the absence of a systemic inflammatory response. In contrast, platelet count significantly increased (*p* = 0.001) with a very large effect (d = 1.68), while changes in mean platelet volume were not statistically significant. All hematological values remained within reference physiological ranges.

## 4. Discussion

The primary objective of this study on nine subjects, which should certainly be considered with caution due to the number of subjects, was to investigate how a five-week progressive application of an elevation mask during high-intensity interval training affects physiological, hematological, and body-composition parameters in recreationally active participants. The most important findings indicate pronounced hematological and moderate body-composition adaptations, with changes observed within a relatively short training period. At the hematological level, an approximately 9.9% increase in erythrocyte count was recorded, comparable to increases described in studies of traditional moderate-altitude training. At the same time, moderate increases in hemoglobin concentration (1.4%) and erythrocyte indices (MCV and MCH) were observed, suggesting favorable morphological changes in the erythroid lineage. Regarding body composition, a slight but consistent reduction in fat tissue was observed alongside minimal changes in fat-free mass, suggesting preferential fat mobilization without a pronounced catabolic effect on muscle tissue.

The intriguing aspect of these results lies in the fact that they were achieved despite the absence of true hypoxia, with SpO_2_ values during tests remaining in the normoxic range (>95%). This raises questions about additional mechanisms that, alongside reduced partial pressure of oxygen, may contribute to increased erythropoiesis in various altitude and hypoxic protocols.

Comparison of the obtained hematological changes with findings from altitude training shows a clear similarity in direction and intensity of adaptation, with a key difference in the mechanism of action. Traditional protocols of living or training at altitude (2000–3000 m) induce hematological changes primarily through reduced partial pressure of oxygen in inspired air, activating HIF-1α signaling and promoting erythropoiesis [17]. Such protocols, however, require specific conditions and longer exposure times.

In this study, a similar pattern of hematological adaptation was achieved without clinically significant hypoxemia, suggesting the existence of different, likely complementary physiological mechanisms. Increased respiratory resistance during ETM use enhances respiratory muscle work, while high-intensity interval training creates strong metabolic stress accompanied by an increased production of lactate, adenosine, and reactive oxygen species. These metabolic factors are known modulators of HIF-1α signaling and can stimulate erythropoiesis even under normoxic conditions, acting as a kind of “hypoxia-equivalent” stimulus.

This combination of mechanical and metabolic loading can, at least partially, reproduce certain adaptive effects of traditional hypoxic exposure without requiring reduced oxygen availability. From a practical standpoint, these findings suggest that specific hematological benefits of altitude training can be achieved under standard conditions using affordable and accessible equipment, which is particularly important for recreational athletes and facilities with limited resources.

The five-week progressive application of an elevation training mask (ETM) during high-intensity interval training (HIIT) induced significant hematological adaptations in recreationally active participants, including significant increases in erythrocyte count (RBC), hemoglobin concentration (Hb), hematocrit (Hct), mean corpuscular volume (MCV), and mean corpuscular hemoglobin (MCH) after the protocol. Such a pattern of changes indicates stimulation of an erythropoietic response, most commonly associated in the literature with hypoxic stimuli.

In this study, these adaptations were achieved using ETM, which mechanically restricts ventilation to simulate conditions of staying at altitudes ranging from approximately 900 to 3600 m, depending on the filter used. Ventilation restriction leads to increased work of the respiratory muscles, including the diaphragm and intercostal muscles (estimated strength increase of 20–50%), while simultaneously reducing minute ventilation (VE) by approximately 10–20% during high-intensity intervals. Such a pattern of ventilatory and metabolic loading can, particularly during repeated intervals, result in the accumulation of functional hypoxic stimuli, which, at the organism level, manifests as reduced oxygenation efficiency and a potential stimulus for the activation of mechanisms involved in erythropoiesis [1].

Consequently, alveolar PO_2_ decreases by 5–10 mmHg (from ~110 to 100 mmHg), and arterial PaO_2_ decreases proportionally because the lungs cannot compensate for the increased oxygen demand of the organism, especially when HIIT is performed with ETM obstructing fresh air delivery [1]. Thus, with a difference between alveolar and arterial pressure of about 10 mmHg, blood oxygen saturation (SpO_2_) decreases. On the steep portion of the oxygen–hemoglobin dissociation curve below 90% saturation, each 1% drop in SpO_2_ corresponds to a 1.5–4 mmHg drop in PaO_2_, further accentuated by the Bohr effect from lactate [12]. Therefore, ETM does not induce the deep hypoxia that occurs at high altitudes, because the mask does not change the oxygen fraction in the air (remains 21%), but only restricts ventilation, creating “functional hypoxia” only under exercise loads above a critical PaO_2_ of ~60 mmHg—sufficient for renal detection and subsequent physiological adaptations without the risk of acute mountain sickness [1].

These mechanisms are supported by several studies documenting improvements in hematological parameters with ETM-HIIT, particularly in recreational athletes and sportsmen. Abouzeid et al. (2023), in their eight-week randomized controlled study on university athletes, showed significant increases in hemoglobin and hematocrit in the experimental group using ETM, along with pronounced improvements in maximal oxygen uptake and lung function compared to the control group [12]. They concluded that ETM-HIIT acts as an effective simulator of respiratory training that induces erythropoietic adaptations similar to altitude conditions, directly supporting the results of our pilot study that cumulative hypoxic dose can improve oxygen transport even in recreational athletes [12]. Similarly, Arumugam et al. (2023) confirmed our findings by demonstrating that HIIT with ETM in recreational athletes induces significant increases in hemoglobin between baseline and final measurements, achieving oxygen transport and heart rate variability comparable to altitude training [19]. The researchers emphasized that the mask effectively stimulates hematological changes through functional hypoxia, confirming that similar protocols in recreational athletes lead to real physiological adaptations [20]. Furthermore, Jerves-Jerves-Donoso et al. (2025) also confirmed the benefits of ETM training in a 12-week CrossFit protocol, where significant improvements in hemoglobin, hematocrit, and erythrocyte count were recorded after the protocol in recreational participants [21].

Therefore, the significant hematological benefits observed in our study after a five-week progressive ETM-HIIT protocol in recreationally active participants indicate strong erythropoietic adaptation. This study points to clearly induced cumulative functional hypoxia, where ventilatory obstruction by the mask reduces minute ventilation and respiratory volume, thereby creating mild alveolar hypoventilation with hypercapnia. Such conditions may create key physiological prerequisites for erythropoietin stimulation, including the activation of hypoxia-inducible factors in the kidneys, without exposing participants to severe hypoxia or the risk of acute mountain sickness. ETM-HIIT thus emerges as an effective, accessible alternative to altitude training for improving oxygen transport in active recreational athletes, warranting further extensive research to confirm all adaptive factors.

Repeated-measures ANOVA (analysis of variance) showed a significant main effect of time on peripheral oxygen saturation, with systematically lower post-test values compared to pre-test throughout the protocol. The significant effect of changing ETM filters across weeks indicates a progressive reduction in SpO_2_ with increasing filter resistance (900–3600 m simulated altitude), particularly from the third to the fifth week. Similarly, maximal heart rate (HRmax) showed a decline during the intervention, with the lowest values in the final weeks, suggesting cardiovascular system adaptation to cumulative respiratory resistance and hypoxemia. These findings confirm that progressive filter changes induce targeted hypoxemia (SpO_2_ drop) alongside a more economical cardiac response (reduced HRmax).

The decline in heart rate during HIIT with an elevation mask, despite high-intensity exercise, occurs after 3–5 weeks of adaptation. Initially, progressive filters (900→3600 m) restrict respiratory volume (from 2.5 to 2 L) and minute ventilation (from 100 to 80 L/min), causing the heart to compensate with high HR (e.g., 175 bpm). After diaphragm strengthening by 20–50%, respiratory volume returns to ~2.3 L, and cardiac output increases by 10–14%, making the lungs more efficient and reducing heart rate (e.g., to 160 bpm for the same cardiac output due to increased stroke volume). Simultaneously, mild hypoxemia (SpO_2_ 92–95%) stimulates carotid chemoreceptors, activating vagal tone and reducing sympathetic activity, which improves heart rate variability by 15–20%—similar to altitude acclimatization—while the central nervous system habituates to the effort, lowering perceived exertion (RPE) and inhibiting unnecessary HR increases. Thus, the heart becomes more economical as the lungs perform better under preserved hypoxia [1,19].

Our study documents mild but consistent anthropometric changes during the five-week ETM-HIIT protocol in recreational athletes. BMI decreased from 24.20 ± 3.48 to 23.77 ± 3.36 kg/m^2^, accompanied by reductions in fat percentage, absolute fat mass, and visceral fat, with only minor decreases in fat-free and bone mass and a slight reduction in basal metabolic rate without changes in metabolic age. Parameter variability and distributions remained stable, indicating homogeneity of the effect in the moderately trained group.

These results indicate a specific reduction in fat tissue without a significant loss of muscle mass, supporting the efficiency of ETM in improving body composition through a combination of hypoxia-induced lipolysis and ventilatory efficiency similar to altitude training effects. Mild hypoxemia induced by ETM physiologically activates the HIF-1α pathway, which promotes fat breakdown (lipolysis) in adipocytes via increased expression of the hormone-sensitive lipase (HSL) enzyme and release of free fatty acids (FFAs). HSL is a key regulator of lipolysis in adipocytes, hydrolyzing triglycerides into FFA and glycerol. In hypoxia, the HIF-1α transcription factor induces its expression and phosphorylation via PKA/cAMP pathways (activated by catecholamines during hypoxic stress), translocating HSL to lipid droplets and thereby increasing fat breakdown more than in normoxia. FFAs are then more efficiently transported to muscles due to increased albumin and hematological adaptations, where β-oxidation generates energy, ultimately resulting in reduced fat tissue [18].

Our results are consistent with the study by Camacho-Cardenosa et al. (2018), who conducted a randomized double-blind study on 82 participants divided into four groups (aerobic interval training in hypoxia, aerobic interval training in normoxia, sprint interval training in hypoxia, sprint interval training in normoxia) over 12 weeks [22]. The study showed that hypoxic groups exhibited superior changes in body composition compared to normoxic groups, with significant reductions in fat percentage, increases in muscle mass, and enhanced fatty acid oxidation [23]. Furthermore, HIF-1α is a key transcription factor stabilized under mild hypoxia by inhibition of PHD enzymes, thereby activating lipases in adipocytes and increasing basal lipolysis by 20–50%, similar to altitude acclimatization effects. Therefore, in the context of our ETM-HIIT study, we can speculate that progressive hypoxemia simulates local deoxygenation, inducing the HIF-1α cascade that releases FFA for β-oxidation in muscles, potentially directly explaining the selective reduction in BMI, fat percentage, visceral fat, and absolute fat mass without significant loss of fat-free mass [22,24].

Despite the relevant findings, this study has several limitations that should be considered when interpreting the results. First, the relatively small number of participants limits statistical power and reduces the possibility of generalizing the results to a broader population. Such a limitation is common in studies involving hypoxic or simulated altitude protocols, where logistical and organizational demands often result in smaller samples. Furthermore, the lack of a control group prevents clear comparison of the effects of the intervention from potential influences of normoxic conditions, as well as other factors such as individual variations in training status, adaptive capacity, and physiological responses. An additional limitation is the non-inclusion of direct measurements of molecular indicators of hypoxic adaptation, such as HIF-1α and erythropoietin concentrations, which limits the deeper interpretation of mechanisms responsible for the observed hematological changes.

Additionally, the motivational status of participants during the intervention and testing protocols was not systematically assessed. Although all participants were informed about the procedure and encouraged to give maximal effort, variable levels of motivation cannot be excluded, which is particularly important in high-intensity protocols where volitional effort can significantly influence performance and physiological response.

Finally, dietary habits and the hydration status of participants were neither controlled nor analyzed in detail, although it is known that nutritional status, iron intake, total energy intake, and hydration can significantly affect hematological indicators and adaptive processes.

Future research should therefore include larger samples, appropriate control groups, objective biomarkers of hypoxic adaptation, and systematic monitoring of motivational, nutritional, and hydration factors to enable more precise interpretation of results and their broader applicability.

## 5. Conclusions

This pilot study suggests that a five-week HIIT program including intermittent use of ETMs is associated with physiological adaptations that in some aspects resemble those observed in altitude-related training, as reported in previous studies. Improvements were recorded in hematological parameters, including increased erythrocyte count, hemoglobin concentration, and hematocrit, which may reflect adaptive changes in oxygen transport capacity and erythropoietic regulation. Furthermore, these changes were accompanied by a tendency toward reduced heart rate responses during the progressive protocol with ETMs, potentially reflecting alterations in autonomic regulation and cardiorespiratory efficiency under increased respiratory load, rather than direct effects of systemic hypoxia. However, due to the pilot nature of this study and the absence of a control group, the observed adaptations cannot be attributed exclusively to the use of ETMs. It is plausible that some of the reported changes, particularly reductions in body fat and increases in erythrocyte-related variables, may also result from the intensive HIIT stimulus alone. Functional hypoxia induced by ETMs may contribute to the modulation of metabolic processes, possibly mediated by hypoxia-sensitive pathways such as HIF-1α, but these interpretations should be considered preliminary. Consequently, while the present findings support the feasibility of ETM-based protocols as an adjunct in sports medicine and endurance-oriented training, their specific physiological contribution cannot be established within the present study design. Therefore, future randomized controlled studies with larger sample sizes and appropriate control conditions are required to clarify causal mechanisms and to confirm the long-term relevance of the observed adaptations.

## Figures and Tables

**Table 1:** Descriptive statistics of anthropometric, body composition, and hematological variables at baseline (IN) and after the five-week intervention (FIN).

Variable	M ± SD (IN)	M ± SD (FIN)	Min–Max (IN)	Min–Max (FIN)
BMI	24.20 ± 3.48	23.77 ± 3.36	18.60–29.80	18.00–29.00
FAT P	16.86 ± 6.27	16.46 ± 6.37	8.00–27.80	7.50–27.80
FAT M	14.37 ± 6.93	13.87 ± 6.69	4.60–27.40	5.40–27.20
FFM	66.64 ± 8.69	65.89 ± 9.27	52.50–81.00	50.20–81.00
VFatL	3.33 ± 2.35	3.00 ± 2.18	1.00–8.00	1.00–7.00
MetaAge	21.22 ± 11.5	20.78 ± 11.5	12.00–38.00	12.00–38.00
FFM	66.66 ± 8.69	65.60 ± 8.52	52.50–81.00	51.30–78.90
BoneM	3.22 ± 0.46	2.94 ± 0.49	2.60–4.00	2.00–3.60
PhysRate	34.22 ± 11.77	33.78 ± 10.00	16.00–53.00	16.00–44.00
BMR	8.15 ± 1.19	7.96 ± 1.27	6.52–10.16	6.23–9.90
Phase	6.82 ± 1.03	6.90 ± 0.63	4.50–8.10	6.00–7.90
ERIT	5.22 ± 0.43	5.74 ± 0.66	4.53–5.86	5.06–6.91
HEMO	155.0 ± 9.12	157.1 ± 8.87	143.0–168.0	144.0–170.0
HEMA	402.7 ± 154.1	357.4 ± 203.5	0.50–502.0	0.46–499.0
MCV	86.61 ± 1.85	87.89 ± 1.51	83.00–89.80	84.80–89.50
MCH	28.36 ± 1.97	30.20 ± 1.49	23.30–29.80	28.60–33.90
MCHC	337.0 ± 8.96	336.4 ± 5.98	326.0–355.0	326.0–344.0
RDW	13.73 ± 0.82	13.73 ± 0.46	12.30–14.30	13.00–14.40
LEUK	7.09 ± 2.13	7.14 ± 1.87	4.60–11.50	5.00–11.80
TROMB	257.8 ± 59.90	264.3 ± 60.34	171.0–336.0	175.0–342.0
MPV	7.48 ± 0.85	7.60 ± 0.69	6.20–9.40	7.20–9.40
CaO_2_	206.8 ± 11.52	209.1 ± 11.68	190.6–223.9	193.9–228.9

**Table 2:** Results of paired-sample *t*-tests and effect sizes for anthropometric, body-composition, and hematological variables at baseline and final measurement.

Variable	AS ± SD (IN)	AS ± SD (FIN)	t	df	*p*	Cohen’s	SE Cohen’s d
BMI	24.20 ± 3.476	23.77 ± 3.363	2.316	8	0.049	0.772	0.061
FAT P	16.86 ± 6.266	16.46 ± 6.370	3.236	8	0.012	1.079	0.024
FAT M	14.37 ± 6.925	13.87 ± 6.690	2.268	8	0.053	0.756	0.034
FFM	66.64 ± 8.686	65.89 ± 9.265	3.063	8	0.016	1.021	0.021
VFatL	3.333 ± 2.345	3.000 ± 2.179	2.000	8	0.081	0.667	0.077
MetaAge	21.22 ± 11.5	20.78 ± 11.5	1.835	8	0.104	0.612	0.024
FFM	66.66 ± 8.691	65.60 ± 8.518	4.104	8	0.003	1.368	0.041
BoneM	3.222 ± 0.460	2.944 ± 0.488	3.054	8	0.016	1.018	0.235
PhysRate	34.22 ± 11.77	33.78 ± 9.997	0.310	8	0.765	0.103	0.121
BMR	8.15 ± 1.189	7.956 ± 1.268	2.479	8	0.038	0.826	0.070
Phase	6.822 ± 1.028	6.900 ± 0.630	−0.407	8	0.695	−0.136	0.172
ERIT	5.222 ± 0.430	5.742 ± 0.664	−3.441	8	0.009	−1.147	0.312
HEMO	155.0 ± 9.124	157.1 ± 8.866	−4.642	8	0.002	−1.547	0.074
HEMA	0.460 ± 0.039	0.486 ± 0.027	−3.478	8	0.008	−1.159	0.246
MCV	86.61 ± 1.849	87.89 ± 1.514	−3.399	8	0.009	−1.133	0.275
MCH	28.36 ± 1.974	30.20 ± 1.488	−2.466	8	0.039	−0.822	0.493
MCHC	337.0 ± 8.958	336.4 ± 5.981	0.198	8	0.848	0.066	0.359
RDW	13.73 ± 0.819	13.73 ± 0.456	−3.763	8	0.006	−1.254	0.367
LEUK	7.089 ± 2.129	7.144 ± 1.874	−0.115	8	0.911	−0.038	0.238
TROMB	257.8 ± 59.90	264.3 ± 60.34	−5.032	8	0.001	−1.677	0.033
MPV	7.478 ± 0.853	7.600 ± 0.691	−0.753	8	0.473	−0.251	0.203

## Data Availability

The original contributions presented in this study are included in the article. Further inquiries can be directed to the corresponding author.
